# Supplementary material for: Infant cries convey both stable and dynamic information about age and identity
Source: Commun Psychol. 2023 Oct 2;1:26. doi: 10.1038/s44271-023-00022-z (PMC11332224; doi:10.1038/s44271-023-00022-z)
Supplement: Supplementary file 1 — Supplementary Information [file 44271_2023_22_MOESM1_ESM.pdf]

Supplementary Table 1 for:

Infant cries convey both stable and dynamic information about age and identity

Marguerite Lockhart-Bouron[1#], Andrey Anikin[2,3#], Katarzyna Pisanski[2,4#], Siloé Corvin[2,5], Clément Cornec[2], Léo Papet[2], Florence Levréro[2], Camille Fauchon[5], Hugues Patural[1§], David Reby[2,6§], Nicolas Mathevon[2,6,7§*]

[1]Neonatal and Pediatric Intensive Care Unit, SAINBIOSE laboratory, Inserm, University Hospital of Saint-Etienne, University of Saint-Etienne, Saint-Etienne, France.

[2]ENES Bioacoustics Research Laboratory, CRNL, CNRS, Inserm, University of Saint-Etienne, Saint-Etienne, France.

[3]Division of Cognitive Science, Lund University, Lund, Sweden.

[4]Laboratoire Dynamique du Langage DDL, CNRS, University of Lyon 2, Lyon, France.

[5]Central Integration of Pain - Neuropain Laboratory, CRNL, CNRS, Inserm, UCB Lyon 1, University of Saint-Etienne, Saint-Etienne, France.

[6]Institut Universitaire de France, Paris, France.

[7]Ecole Pratique des Hautes Etudes, PSL Research University, Paris, France.

# These authors contributed equally

§ These authors jointly supervised this work

Supplementary Table 1. Number of cry sequences and extracted cries per baby, age, and cry cause.

Baby ID	Sex	Cry sequences	Cry sequences by age (0.5/1.5/2.5/3.5 m)	Cry sequences by cause (discomfort / hunger / isolation)	Cries	Cries by age (0.5/1.5/2.5/3.5 m)	Cries by cause (discomfort / hunger / isolation)
BM29	M	26	0 / 11 / 11 / 4	11 / 5 / 10	2327	0 / 896 / 1157 / 274	972 / 589 / 766
BR07	M	25	12 / 8 / 5 / 0	6 / 14 / 5	750	382 / 252 / 116 / 0	126 / 506 / 118
BR15	F	24	7 / 6 / 11 / 0	3 / 16 / 5	1060	161 / 224 / 675 / 0	32 / 736 / 292
BS03	F	13	9 / 0 / 3 / 1	2 / 4 / 7	645	382 / 0 / 262 / 1	130 / 113 / 402
CA12	F	46	12 / 17 / 10 / 7	24 / 15 / 7	2228	545 / 721 / 522 / 440	1323 / 481 / 424
GL13	M	33	11 / 4 / 11 / 7	8 / 18 / 7	1729	790 / 201 / 327 / 411	959 / 450 / 320
HA22	F	4	0 / 1 / 2 / 1	2 / 0 / 2	327	0 / 7 / 128 / 192	76 / 0 / 251
KA02	M	22	8 / 7 / 0 / 7	2 / 11 / 9	1657	451 / 674 / 0 / 532	516 / 582 / 559
LC10	F	23	9 / 10 / 4 / 0	9 / 5 / 9	593	175 / 344 / 74 / 0	166 / 138 / 289
LC26	M	47	21 / 14 / 12 / 0	9 / 22 / 16	2796	1281 / 918 / 597 / 0	488 / 1004 / 1304
ML21	M	16	0 / 12 / 4 / 0	8 / 4 / 4	465	0 / 390 / 75 / 0	118 / 96 / 251
MR30	F	43	13 / 13 / 17 / 0	6 / 19 / 18	2775	1115 / 895 / 765 / 0	421 / 1394 / 960
PA08	M	18	8 / 7 / 3 / 0	0 / 12 / 6	4140	2778 / 643 / 719 / 0	0 / 1649 / 2491
PA27	F	16	7 / 4 / 5 / 0	3 / 8 / 5	1261	376 / 321 / 564 / 0	87 / 695 / 479
PB24	M	29	0 / 17 / 12 / 0	11 / 5 / 13	2796	0 / 791 / 2005 / 0	1467 / 160 / 1169
PE18	M	16	0 / 8 / 3 / 5	3 / 6 / 7	1310	0 / 853 / 305 / 152	243 / 141 / 926
PJ14	F	21	10 / 8 / 3 / 0	6 / 11 / 4	864	381 / 410 / 73 / 0	418 / 218 / 228
RB01	M	44	11 / 16 / 13 / 4	10 / 13 / 21	2707	631 / 1401 / 354 / 321	280 / 660 / 1767
SA20	M	31	7 / 9 / 9 / 6	7 / 14 / 10	2937	679 / 743 / 764 / 751	808 / 942 / 1187
SB23	M	18	10 / 7 / 1 / 0	7 / 8 / 3	414	108 / 164 / 142 / 0	132 / 256 / 26
TA05	F	27	6 / 8 / 7 / 6	4 / 19 / 4	1611	423 / 435 / 434 / 319	71 / 1339 / 201
TL16	M	18	0 / 9 / 9 / 0	7 / 6 / 5	417	0 / 193 / 224 / 0	248 / 93 / 76
TM04	M	29	5 / 6 / 9 / 9	11 / 9 / 9	2709	329 / 503 / 774 / 1103	1270 / 573 / 866
XM17	F	23	0 / 12 / 7 / 4	5 / 11 / 7	683	0 / 420 / 179 / 84	146 / 280 / 257
Total (24 babies)		612	166 / 214 / 171 / 61	164 / 255 / 193	39201	10987 / 12399 / 11235 / 4580	10497 / 13095 / 15609
