# Supplementary material for: Infant cries convey both stable and dynamic information about age and identity
Source: Commun Psychol. 2023 Oct 2;1:26. doi: 10.1038/s44271-023-00022-z (PMC11332224; doi:10.1038/s44271-023-00022-z)
Supplement: Supplementary file 2 — Supplementary Data [file 44271_2023_22_MOESM2_ESM.pdf]

Supplementary Data for:

Infant cries convey both stable and dynamic information about age and identity

Marguerite Lockhart-Bouron[1#], Andrey Anikin[2,3#], Katarzyna Pisanski[2,4#], Siloé Corvin[2,5], Clément Cornec[2], Léo Papet[2], Florence Levréro[2], Camille Fauchon[5], Hugues Patural[1§], David Reby[2,6§], Nicolas Mathevon[2,6,7§*]

[1]Neonatal and Pediatric Intensive Care Unit, SAINBIOSE laboratory, Inserm, University Hospital of Saint-Etienne, University of Saint-Etienne, Saint-Etienne, France.

[2]ENES Bioacoustics Research Laboratory, CRNL, CNRS, Inserm, University of Saint-Etienne, Saint-Etienne, France.

[3]Division of Cognitive Science, Lund University, Lund, Sweden.

[4]Laboratoire Dynamique du Langage DDL, CNRS, University of Lyon 2, Lyon, France.

[5]Central Integration of Pain - Neuropain Laboratory, CRNL, CNRS, Inserm, UCB Lyon 1, University of Saint-Etienne, Saint-Etienne, France.

[6]Institut Universitaire de France, Paris, France.

[7]Ecole Pratique des Hautes Etudes, PSL Research University, Paris, France.

# These authors contributed equally

§ These authors jointly supervised this work

Supplementary Data. *EnesBabyCries1* acoustic databank.

The cries collected for this study are now available in the form of a new cry databank (*EnesBabyCries1*).
This archive contains two sets of audio recordings in WAV format:

- *EnesBabyCries1A* contains the sequences of recordings from step 3 (i.e., after removal of noisy parts).

- *EnesBabyCries1B* contains the cries from step 6 (after segmentation and selection of the cries; these cries are the ones used in the present research).

This databank is anonymized. No clues to the identity of the baby have been retained in the recordings. The metadata accompanying the cries are: 1) the age of the recorded baby; 2) the baby's biological sex; 3) the cause of the cry as stated by the parent; 4) the parental action that ended the cry. We obtained parental permission to make these recordings and information public.

The *EnesBabyCries1* databank is available here: **https://osf.io/ru7na/.**
