# Supplementary material for: Infant cries convey both stable and dynamic information about age and identity
Source: Commun Psychol. 2023 Oct 2;1:26. doi: 10.1038/s44271-023-00022-z (PMC11332224; doi:10.1038/s44271-023-00022-z)
Supplement: Supplementary file 4 — Description of Additional Supplementary Files [file 44271_2023_22_MOESM4_ESM.pdf]

Description of Additional Supplementary Files

File Name: Supplementary Data

Description: Description of the EnesBabyCries1 acoustic databank.
